# Electrical Control in Neurons by the Ketogenic Diet

**DOI:** 10.3389/fncel.2018.00208

**Published:** 2018-07-16

**Authors:** Nagisa Sada, Tsuyoshi Inoue

**Affiliations:** ^1^Department of Biophysical Chemistry, Graduate School of Medicine, Dentistry and Pharmaceutical Sciences, Okayama University, Okayama, Japan; ^2^Department of Hygiene, Kawasaki Medical School, Kurashiki, Japan

**Keywords:** ketogenic diet, ketone body, glucose, lactate, epilepsy, antiepileptic drug, electrophysiology

## Abstract

The ketogenic diet is used as a diet treatment for drug-resistant epilepsy, but there are no antiepileptic drugs based on the ketogenic diet. The ketogenic diet changes energy metabolites (ketone bodies, glucose and lactate) in the brain, which consequently changes electrical activities in neurons and ultimately suppresses seizures in epileptic patients. In order to elucidate the antiseizure effects of the ketogenic diet, it is important to clarify the mechanism by which these metabolic changes are converted to electrical changes in neurons. In this review, we summarize electrophysiological studies focusing on electrical control in neurons by the ketogenic diet. Recent studies have identified electrical regulators driven by the ketogenic diet: ion channels (ATP-sensitive K^+^ channels and voltage-dependent Ca^2+^ channels), synaptic receptors (AMPA-type glutamate receptors and adenosine A_1_ receptors), neurotransmitter transporters (vesicular glutamate transporters), and others (BCL-2-associated agonist of cell death and lactate dehydrogenase). Thus, the ketogenic diet presumably elicits neuronal inhibition via the combined actions of these molecules. From the viewpoint of drug development, these molecules are valuable as targets for the development of new antiepileptic drugs. Drug therapy to mimic the ketogenic diet may be feasible in the future, through the combination of multiple antiepileptic drugs targeting these molecules.

## Introduction

Neurons, which are connected by synapses, exhibit electrical activities in the living brain. The electrical activities in neurons are generated by ion channels and synaptic receptors. Although these electrical activities are essential for normal brain function, they become excessive in the brains of epileptic patients. Therefore, medicines for epilepsy have to suppress the neuronal hyperexcitation, and currently-used antiepileptic drugs have been designed to act on ion channels and synaptic receptors (Meldrum and Rogawski, 2007). For example, carbamazepine and lamotrigine inhibit voltage-dependent Na^+^ channels, gabapentin inhibits the α2δ subunits of L-type Ca^2+^ channels, phenobarbital activates GABA_A_ receptors, and perampanel modulates AMPA-type glutamate receptors (reviewed in Bialer and White, 2010).

However, these antiepileptic drugs are not effective for all epileptic patients. Approximately 1% of the world’s population suffer from epilepsy, and one-third of epileptic patients are resistant to currently-available antiepileptic drugs (Kwan and Brodie, 2000). It should be noted that the diet treatment using the ketogenic diet is effective for some patients with drug-resistant epilepsy (Freeman et al., 1998; Neal et al., 2008). However, since the ketogenic diet is an unbalanced diet consisting of high-fat and low-carbohydrate, new medicines based on the ketogenic diet will be useful for the treatment of drug-resistant epilepsy. To address this issue, recent studies have elucidated the antiseizure mechanisms of the ketogenic diet at the molecular level (reviewed in Lutas and Yellen, 2013; Boison, 2017; Rho, 2017; Simeone et al., 2018). Since these reviews have introduced many antiseizure mechanisms associated with the metabolic, electrical, epigenetic and inflammatory changes induced by the ketogenic diet, we do not summarize these mechanisms thoroughly in the present review. We here summarize electrophysiological studies that focus on electrical control in neurons by the ketogenic diet.

From the viewpoint of drug development, the molecules involved in the ketogenic diet are valuable as “target molecule” to explore candidates of new antiepileptic drugs. Toward drug development, we then identify small chemical compounds acting on the target molecule, called as “seed compound” for screening from compound libraries. We finally identify drug candidates by compound screening using the target molecule and seed compound. Thus, the identification of molecules involved in the ketogenic diet is the first step toward the development of new antiepileptic drugs for drug-resistant epilepsy.

## Ketogenic Diet for Drug-Resistant Epilepsy

The ketogenic diet treatment was developed in the 1920s, and its concept dates back to biblical times (Wheless, 2008). The diet treatment was originally developed by Dr. Wilder at the Mayo Clinic in 1921, and its modified version using a medium-chain triglyceride (MCT) diet was developed in the 1970s (Huttenlocher et al., 1971). These ketogenic diets are high-fat and low-carbohydrate diets, which produce ketone bodies (β-hydroxybutyrate and acetoacetate) by the liver. Ketone bodies are then delivered to the brain and used as alternative energy sources to glucose. The ketogenic diet not only increases ketone bodies, but also mildly decreases blood glucose levels in epileptic patients (Huttenlocher, 1976). These two metabolic changes are now recognized to exert antiseizure effects (reviewed in Rho, 2017). The direct actions of ketone bodies were recently reviewed in detail (Simeone et al., 2018).

Historically, although the ketogenic diet treatment was developed in 1921, its use in clinical setting decreased after the development of the antiepileptic drug diphenylhydantoin (phenytoin) in 1938 (Merritt and Putnam, 1938). However, the diet treatment re-attracted attention in the 1990s because it was shown to be effective for patients with drug-resistant epilepsy (Freeman et al., 1998). The ketogenic diet treatment is now used in many countries worldwide (Kossoff and McGrogan, 2005).

The efficacy of the ketogenic diet for drug-resistant epilepsy has been confirmed in many clinical studies. Randomized clinical trials revealed that approximately 40% of pediatric patients with drug-resistant epilepsy were controlled by the ketogenic diet (Neal et al., 2008) and that the efficacy was similar between the classical and MCT versions of the ketogenic diet (Neal et al., 2009). The ketogenic diet was effective for patients with Dravet syndrome (Caraballo et al., 2005), a severe childhood epilepsy with high mortality (Sakauchi et al., 2011). In addition to childhood epilepsy, growing evidence supports that the ketogenic diet is also effective for adult patients with intractable epilepsy (Klein et al., 2014; Liu et al., 2018).

## Molecules for Electrical Control by the Ketogenic Diet

**Figure 1:**
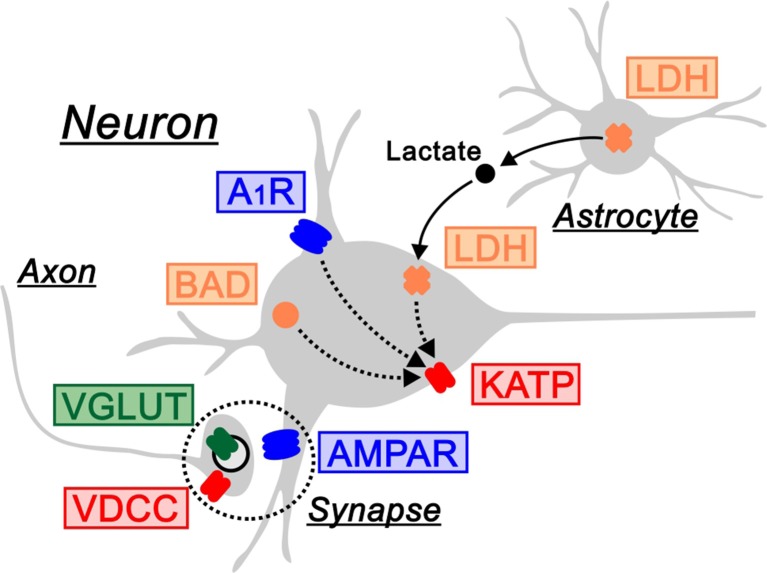
Electrical regulators driven by the ketogenic diet. The ketogenic diet is associated with the following molecules that regulate electrical activities in neurons: ATP-sensitive K^+^ channels (KATP) and voltage-dependent Ca^2+^ channels (VDCC) as ion channels in *red*, AMPA-type glutamate receptors (AMPAR) and adenosine A_1_ receptors (A_1_R) as synaptic receptors in *blue*, vesicular glutamate transporters (VGLUT) as neurotransmitter transporters in *green*, and BCL-2-associated agonist of cell death (BAD) and lactate dehydrogenase (LDH) as other molecules in *orange*. LDH is located in the astrocyte-neuron lactate shuttle (arrows). A_1_R, BAD and LDH regulate electrical activities via K_ATP_ channels (dotted arrows). Synapse is indicated by dotted circles.

As described above, the ketogenic diet is now established as a treatment for patients with drug-resistant epilepsy. Since seizures are elicited by the hyperexcitation of electrical activities in neurons, it is mechanistically presumed that the ketogenic diet acts on the molecules generating electrical currents, such as ion channels and synaptic receptors. This issue can be directly addressed using electrophysiological techniques in rodents. We therefore introduce electrophysiology-based studies, in which the following seven molecules have been identified as regulators of neuronal electrical activities by the ketogenic diet (Figure 1).

### ATP-Sensitive K^+^ Channels

The ketogenic diet consists of high-fat and low-carbohydrate, and mainly elicits two metabolic changes in blood: increases in ketone bodies and decreases in glucose (Bough et al., 2006). In rodent studies, the ketogenic diet was shown to increase the blood plasma level of β-hydroxybutyrate to 1–8 mM (Bough and Eagles, 1999; Bough et al., 1999, 2006), which is similar to that in humans (Neal et al., 2009). Using electrophysiological techniques, Yellen and colleagues found adenosine 5’-triphosphate (ATP)-sensitive K^+^ channels (K_ATP_ channels) as the molecule that ketone bodies acted on (Ma et al., 2007). K_ATP_ channels are known to be metabolic sensors that regulate electrical activities, which are blocked by intracellular ATP. Ma et al. (2007) proposed that ketone bodies increase intracellular global ATP, but decrease glycolytic ATP production, which leads to decreases in ATP near the plasma membrane, the opening of K_ATP_ channels, and reductions in the firing rate of neurons. Consistent with this, the opening of K_ATP_ channels by ketone bodies was directly demonstrated by single channel recordings (Tanner et al., 2011).

### Voltage-Dependent Ca^2+^ Channels

Voltage-dependent Ca^2+^ channels (VDCCs) in presynaptic terminals are known to be essential for synaptic transmission (Zucker and Regehr, 2002; Catterall et al., 2013). By using patch-clamp recordings from hippocampal slices, Inoue and colleagues demonstrated that acetoacetate inhibited Ca^2+^ influx through VDCCs in CA1 pyramidal cells (Kadowaki et al., 2017). They also found that acetoacetate modulated the short-term synaptic plasticity of excitatory postsynaptic currents (EPSCs) in pyramidal cells, showing that it acted on presynaptic VDCCs. Interestingly, acetoacetate reduced EPSCs in slices exhibiting epileptiform activities, but not in normal slices (Kadowaki et al., 2017), suggesting that the effects of acetoacetate preferably emerge in the hyperexcitable state of the brain. Although its VDCC types remain unclear, it is probably mediated by P/Q-type (Cav2.1) or N-type (Cav2.2) Ca^2+^ channels, because these Ca^2+^ channels mainly contribute to synaptic transmission (Catterall et al., 2013). A previous study reported that β-hydroxybutyrate inhibited N-type Ca^2+^ channels in rat sympathetic neurons of the peripheral nervous system (Won et al., 2013).

### Vesicular Glutamate Transporters

Vesicular glutamate transporters (VGLUTs), which fill synaptic vesicles with glutamate, critically regulate excitatory synaptic transmission in the brain (Fremeau et al., 2004). Thus, VGLUTs are also electrical regulators that indirectly affect postsynaptic glutamate receptors. Using a proteoliposome containing purified VGLUTs, Moriyama and colleagues demonstrated that acetoacetate was a specific inhibitor of VGLUTs (Juge et al., 2010). Although VGLUTs were activated by Cl^−^ ions, the elevated VGLUT activities were inhibited by acetoacetate: therefore, they proposed that VGLUTs are oppositely regulated by Cl^−^ ions and acetoacetate. Functionally, acetoacetate inhibited miniature EPSCs in CA1 pyramidal cells of hippocampal slices and suppressed acute seizures in rats *in vivo* (Juge et al., 2010).

### Adenosine A_1_ Receptors

Adenosine is an inhibitory neuromodulator, which acts on four types of adenosine receptors in the brain (A_1_, A_2A_, A_2B_ and A_3_; Dunwiddie and Masino, 2001). Among them, the activation of adenosine A_1_ receptors (A_1_Rs) was shown to suppress chronic seizures in a mouse model of pharmacoresistant mesial temporal lobe epilepsy (Gouder et al., 2003). Boison and colleagues found that the antiseizure effects of the ketogenic diet were not observed in knockout mice of adenosine A_1_Rs (Masino et al., 2011). Furthermore, the antiseizure mechanism is presumably attributed to the decreases in glucose by the ketogenic diet. By using patch-clamp recordings from hippocampal slices, Masino and colleagues revealed that the decreases in glucose hyperpolarized hippocampal pyramidal cells, and this was mediated by adenosine A_1_Rs and K_ATP_ channels (Kawamura et al., 2010). They further found that the decreases in glucose reduced the neural excitability in mice fed the ketogenic diet, and this was also mediated by adenosine A_1_Rs and K_ATP_ channels (Kawamura et al., 2014).

### AMPA-Type Glutamate Receptors

The MCT ketogenic diet is a modified version of the classical ketogenic diet (Huttenlocher et al., 1971), and increases not only ketone bodies but also two fatty acids (octanoic and decanoic acids) in the blood of epileptic patients (Haidukewych et al., 1982; Sills et al., 1986). Octanoic and decanoic acids are straight-chain saturated monocarboxylic acids with 8 and 10 carbons, respectively. These medium-chain fatty acids directly penetrate the blood-brain barrier (Oldendorf, 1973). Walker, Williams, and colleagues reported that decanoic acid, but not octanoic acid, suppressed *in vitro* epileptiform activity in entorhinal cortex-hippocampus slices (Chang et al., 2013). They also elucidated the underlying mechanism, in which decanoic acid reduced EPSCs via AMPA-type glutamate receptors (Chang et al., 2016). By using electrophysiological recordings from an oocyte expression system, they showed that decanoic acid inhibited glutamate-induced currents derived from various types of AMPA receptor subunits (GluA1, GluA1/2 and GluA2/3), and also showed that it was the most sensitive to GluA2/3 (Chang et al., 2016).

### BCL-2-Associated Agonist of Cell Death

BCL-2-associated agonist of cell death (BAD) is a member of the BCL-2 family, which is known to regulate cellular metabolism (Giménez-Cassina and Danial, 2015). The knockout of BAD decreases the ability to use glucose and increases the ability to use β-hydroxybutyrate (Giménez-Cassina et al., 2012), which is similar to metabolism during the ketogenic diet (Bough et al., 2006). Yellen, Danial, and colleagues demonstrated that this metabolic switch by the BAD knockout protected against acute seizures *in vivo* (Giménez-Cassina et al., 2012). Chronic seizures in *Kcna1*-null mice, a mouse model of sudden unexpected death in epilepsy, were also suppressed by the BAD knockout (Foley et al., 2018), as well as by the ketogenic diet (Fenoglio-Simeone et al., 2009; Simeone et al., 2016).

From the viewpoint of electrical control, the antiseizure effects of the BAD knockout are mediated by K_ATP_ channels (Giménez-Cassina et al., 2012; Martínez-François et al., 2018). K_ATP_ channel currents in hippocampal neurons were shown to become larger in BAD-knockout mice, which was blocked by the genetic ablation of Kir6.2 (a pore-forming subunit of K_ATP_ channels) or K_ATP_ channel blockers (Giménez-Cassina et al., 2012). Consistent with this finding, the BAD knockout reduced neuronal excitability and epileptiform activity in brain slices, which was canceled by K_ATP_ channel blockers (Martínez-François et al., 2018). The *in vivo* antiseizure effects of the BAD knockout were also canceled by the genetic ablation of Kir6.2 (Giménez-Cassina et al., 2012). Thus, the BAD → K_ATP_ channel pathway regulates electrical and seizure control by the ketogenic diet. BAD is a unique molecule as electrical regulators by the ketogenic diet, because it mimics two metabolic changes during the ketogenic diet (decreases in glucose and increases in ketone bodies).

### Lactate Dehydrogenase

Glucose is directly transported into neurons and used as an energy source. As an alternative metabolic pathway, glucose is transported into astrocytes and converted to lactate, which is then released to extracellular spaces and transported into neurons: this metabolic pathway is called the astrocyte-neuron lactate shuttle (Bélanger et al., 2011). Lactate is suggested to be a preferred energy source over glucose in the brain (Larrabee, 1995; Smith et al., 2003). This astrocyte-derived lactate regulates electrical activities in neurons (Rouach et al., 2008; Parsons and Hirasawa, 2010). Based on these backgrounds, Inoue and colleagues demonstrated that the astrocyte-neuron lactate shuttle contributes to neuronal inhibition and seizure suppression by the ketogenic diet (Sada et al., 2015). By using slice patch-clamp recordings, they found that ketogenic-like metabolic changes induced hyperpolarization in neurons, which was recovered by the activation of the lactate shuttle. They also found that the inhibition of lactate dehydrogenase (LDH), a metabolic enzyme located in the astrocyte-neuron lactate shuttle, induced hyperpolarization in neurons and suppressed chronic seizures *in vivo* in a mouse model of epilepsy (Sada et al., 2015).

From the viewpoint of electrical control, the neuronal hyperpolarization by the LDH inhibition is mediated by K_ATP_ channels (Sada et al., 2015). They showed that the LDH inhibition induced hyperpolarization, which was recovered by K_ATP_ channel blockers. This hyperpolarization was also recovered by pyruvate, a downstream metabolite of LDH, indicating that it is mediated by pyruvate (Sada et al., 2015). Furthermore, this hyperpolarization was not recovered by an intracellular injection of ATP, suggesting that it is not dependent on ATP (Sada et al., 2015). Taken together, the LDH → pyruvate → K_ATP_ channel pathway regulates electrical and seizure control by the ketogenic diet, which works in an energy-independent manner. LDH is the first metabolic enzyme identified as electrical regulators responsible for the ketogenic diet.

## Drug Development Based on the Ketogenic Diet

The ketogenic diet treatment requires strict dietary control. Since medicines are markedly easier to administer, antiepileptic drugs based on the ketogenic diet will be useful for epileptic patients. Historically, the ketogenic diet treatment developed in the 1920s shifted to drug therapy using phenytoin in the late 1930s (Wheless, 2008).

Several approaches have been reported toward the development of ketogenic diet-based antiepileptic drugs. The first approach is ketone supplementation without dietary control. The oral administration of *R,S*-1,3-butanediol acetoacetate diester, a ketone ester, was shown to increase ketone bodies and decrease glucose in rat blood (D’Agostino et al., 2013; Kesl et al., 2016). These metabolic changes are similar to those observed during the ketogenic diet (Bough et al., 2006). Consistent with these findings, the ketone ester attenuated seizures in a mouse model of Angelman syndrome (Ciarlone et al., 2016) and in a rat model of absence epilepsy (Kovács et al., 2017). This ketone supplementation without dietary control may be useful as a new treatment for epilepsy that mimics the ketogenic diet.

The second approach is the new antiepileptic drug perampanel that was recently developed by a pharmaceutical company. It is designed to act on AMPA-type glutamate receptors (Hanada et al., 2011) and is effective for epileptic patients (French et al., 2012; Krauss et al., 2012). Different lines of basic studies by Walker, Williams, and colleagues demonstrated that decanoic acid, a metabolite of the MCT ketogenic diet, suppressed epileptiform activities via AMPA-type glutamate receptors (Chang et al., 2013, 2016). Thus, these two lines of evidence indicate that perampanel is a clinically-available drug to mimic the mechanism of action of the ketogenic diet.

The third approach originates from the antiepileptic drug stiripentol. Stiripentol has been clinically administrated to epileptic patients with Dravet syndrome (Chiron et al., 2000). It was originally shown to act on the GABAergic system in the brain (Trojnar et al., 2005; Quilichini et al., 2006). Inoue and colleagues reported that stiripentol inhibited LDH, a metabolic enzyme responsible for the antiseizure effects of the ketogenic diet (Sada et al., 2015). They further identified a compound smaller than stiripentol, which retained the ability to inhibit LDH enzyme and suppress seizures *in vivo*. They proposed a strategy for drug development based on the ketogenic diet, that is, targeting LDH enzyme with stiripentol derivatives.

Interestingly, although K_ATP_ channels are directly activated by ketone bodies (Ma et al., 2007; Tanner et al., 2011), it is also indirectly activated by BAD (Giménez-Cassina et al., 2012; Martínez-François et al., 2018) and LDH (Sada et al., 2015). K_ATP_ channels are also involved in neuronal inhibition by adenosine A_1_Rs (Kawamura et al., 2010, 2014; Masino et al., 2011). Thus, K_ATP_ channels are potential target molecules for drug development based on the ketogenic diet. However, it remains unclear whether K_ATP_ channel blockers themselves are appropriate for the drug candidate. For example, K_ATP_ channel blockers act on all types of neurons in the hippocampus (Zawar et al., 1999). In contrast, Sada et al. (2015) showed that the LDH inhibition was able to selectively hyperpolarize excitatory pyramidal cells but did not change inhibitory interneurons in the hippocampus.

Finally, some clinical studies have tested the feasibility of the ketogenic diet for the treatment of other neurological disorders. A clinical trial revealed that a modified version of the ketogenic diet improved core autism features in patients with autism spectrum disorder (Lee et al., 2018), and this was supported by animal studies (Ruskin et al., 2013, 2017). In addition, pilot clinical studies reported that the MCT ketogenic diet improved the cognitive performance of patients with Alzheimer’s disease or mild cognitive impairment (Reger et al., 2004), and also reported that the ketogenic diet improved Parkinson’s disease symptoms (Vanitallie et al., 2005). Although their efficacies have not fully evaluated in detail, further studies may potentially lead to drug development for these neurological disorders.

## Conclusion

The ketogenic diet is effective for patients with drug-resistant epilepsy, and thus, these patients hunger for new antiepileptic drugs based on the ketogenic diet. Since seizures are elicited by the hyperexcitation of electrical activities in neurons, recent studies have uncovered the molecules responsible for the electrical control in neurons by the ketogenic diet. These electrical regulators are ion channels (K_ATP_ channels and VDCCs), synaptic receptors (AMPA-type glutamate receptors and adenosine A_1_Rs), neurotransmitter transporters (VGLUTs), and others (BAD and LDH). Neuronal inhibition and seizure suppression by the ketogenic diet are presumed to be achieved by the combined actions of these molecules. Furthermore, some studies are now attempting to develop antiepileptic drugs targeting these molecules. Drug therapy to mimic the ketogenic diet will be possible in the future, through the combination of multiple antiepileptic drugs targeting these molecules.

## Author Contributions

NS and TI designed the idea and wrote and edited the manuscript.

## Conflict of Interest Statement

The authors declare that the research was conducted in the absence of any commercial or financial relationships that could be construed as a potential conflict of interest.
